# Characterisation of a wild-type influenza (A/H1N1) virus strain as an experimental challenge agent in humans

**DOI:** 10.1186/s12985-015-0240-5

**Published:** 2015-02-03

**Authors:** Jeannette M Watson, James N Francis, Sofie Mesens, Gabriel A Faiman, Jill Makin, Peter Patriarca, John J Treanor, Bertrand Georges, Campbell J Bunce

**Affiliations:** Immune Targeting Systems Ltd, London BioScience Innovation Centre, 2 Royal College Street, London, NW1 0NH UK; SGS LSS, Clinical Research Unit, Antwerpen, Belgium; Jill Makin Consulting Ltd, 7 Cholmondeley Road, West Kirby, Wirral CH48 7HB UK; Biologics Consulting Group, INC, 1317 King Street, Alexandria, VA 22314 USA; University of Rochester Medical Center, 601 Elmwood Avenue, Box 689, Rochester, NY 14642 USA

**Keywords:** Influenza A/H1N1 virus, Challenge agent, Vaccine, Clinical trial

## Abstract

**Background:**

Human challenge models using respiratory viruses such as influenza are increasingly utilised in the development of novel vaccines and anti-viral modalities and can provide preliminary evidence of protection before evaluation in field trials. We describe the results of a clinical study characterising an A/H1N1 influenza challenge virus in humans.

**Methods:**

The challenge agent, influenza A/California/2009 (H1N1), was manufactured under cGMP conditions and characterised in accordance with regulatory guidelines. A dose-ascending open-label clinical study was conducted in 29 healthy young adults screened sero-negative to the challenge strain. Subjects were intranasally inoculated with three increasing doses of virus and physician-reported signs, subjected-reported symptoms, viral shedding and immunological responses were monitored.

**Results:**

A dose-dependent increase in clinical signs and symptoms was observed with 75% of subjects developing laboratory-confirmed illness at the highest inoculum (3.5 × 10^6^ TCID_50_). At the highest dose, physician or subject-reported signs of infection were classified as mild (all subjects), moderate (50%) and severe (16%) with peak symptoms recorded four days after infection. Clinical signs were correlated with nasal mucus weight (*P* < .001) and subject-reported symptoms (*P* < .001). Geometric mean peak viral shedding was log_10_ 5.16 TCID_50_ and occurred three days after inoculation with a median duration of five days. The safety profile was such that physiological responses to viral infection were mainly restricted to the upper airways but were not of such severity to be of clinical concern.

**Conclusions:**

A highly characterised wild-type Influenza A/California/2009 (H1N1) virus manufactured for clinical use was shown to induce a good infectivity profile in human volunteers. This clinical challenge model can be used for evaluating potential efficacy of vaccines and anti-viral therapeutics.

**Trial registration:**

NCT02014870

**Electronic supplementary material:**

The online version of this article (doi:10.1186/s12985-015-0240-5) contains supplementary material, which is available to authorized users.

## Background

Influenza A is a major global health concern with seasonal influenza epidemics affecting 5 to 15% of the population, resulting in severe illness in 3 to 5 million patients and approximately 250,000 to 500,000 deaths per year [1]. Hospitalisation and deaths mainly occur in high-risk groups, in particular children younger than age two, adults age 65 or older, and people of any age with certain medical conditions, such as chronic heart, lung, kidney, liver, blood or metabolic diseases (such as diabetes), or weakened immune systems [2].

Effective control of influenza requires the use of vaccines and antiviral treatments. Whilst traditional influenza vaccines can offer a high level of protection in healthy adults, reduced efficacy is observed in high-risk groups [1]. New strategies are being investigated to develop more effective vaccines against both seasonal and pandemic influenza including universal influenza vaccines [3-5]. Emergence of antiviral resistance amongst circulating influenza strains exemplifies the required development of novel prophylactic and therapeutic strategies [6].

Experimentally infecting healthy volunteers with a well characterised influenza virus provides a unique opportunity to evaluate new intervention strategies under controlled conditions in proof of concept studies. Such trials can establish pharmacological activity in humans and help identify correlates of protection. Data from challenge studies can also contribute to dose selection and understanding of timing of intervention before progressing to larger field-based studies.

Here we describe the characterisation and clinical validation of an influenza A/H1N1 challenge virus, isolated during the 2009 ‘swine-flu’ pandemic, suitable for use in human challenge studies.

## Results

### Clinical study

A total of 29 healthy human volunteers aged between 22 and 45 (median 38) were infected with a live influenza A virus, A/California/2009, in an open-label, dose-escalation clinical study (Table 1). All enrolled volunteers were seronegative to the challenge strain and were isolated in a quarantine unit from 24 hours prior to challenge to 7 days after challenge. Signs and symptoms of influenza were recorded via a targeted physical examination assessed by a physician and subject scoring card respectively. Body temperature was measured every 4 h during the day to assess for presence of fever. Nasal washes were performed daily to determine viral shedding. We initially challenged 5 volunteers with a 1:1000 dilution (Cohort 1) of the virus (~3.5 × 10^4^ TCID_50_), followed by 12 subjects (Cohort 2) at 1:100 dilution (~3.5 × 10^5^ TCID_50_) and a further 12 subjects (Cohort 3) at a 1:10 dilution (~3.5 × 10^6^ TCID_50_). Before progressing to Cohorts 2 and 3, safety and infectivity data was reviewed by a safety review committee.

**Table 1:** **Subject characterisation**

**Parameter**	**Cohort 1**	**Cohort 2**	**Cohort 3**	**All subjects**
	**1/1000 dilution of neat virus**	**1/100 dilution of neat virus**	**1/10 dilution of neat virus**	
	**N = 5**	**N = 12**	**N = 12**	**N = 29**
Age, years; Median (range)	40.0 (39; 45)	35.0 (22; 44)	39.5 (25; 45)	38.0 (22; 45)
Height, cm; Median (range)	165.5 (154; 180)	168.3 (153; 180)	175.0 (155; 184)	173.3 (153; 184)
Weight, kg; Median (range)	62.4 (56; 78)	68.0 (50; 90)	74.2 (50; 97)	68.7 (50; 97)
Sex, n (% Female); Female	2 (40.0)	9 (75.0)	4 (33.3)	16 (55.2)
Race, n (%)				
Asian	1 (20.0)	0	0	1 (3.4)
White	4 (80.0)	12 (100.0)	12 (100.0)	28 (96.6)

N = number of subjects per cohort; n = number of subjects with that observation.

### Safety and tolerability

Viral challenge was expected to induce symptoms/signs typical of influenza infection and were captured as infectivity endpoints in the study and not as adverse events, unless they were graded as severe by either the subject or investigator. Across the study, no serious adverse events (SAEs) were recorded. Additional file 1: Table S1 summarises all treatment-emergent adverse events (TEAE) that were reported during the quarantine phase. 3 (60.0%) subjects of Cohort 1, 6 (50.0%) subjects of Cohort 2, and 5 (41.7%) subjects of Cohort 3 had reported at least one TEAE. In total, 14 subjects (48.3%) reported 34 events. Most TEAEs were mild or moderate in severity. There were no clinically significant or persistent shifts in safety laboratory values reported. One subject in Cohort 2 (202) had an elevated absolute neutrophil count (10.04 × 10^9^/L) and total white cell count (13.9 × 10^9^/L) two days after challenge. Three subjects in Cohort 2 (206, 209, and 211) and 2 subjects in Cohort 3 (301 and 304) experienced elevated C-reactive proteins in the range of 16.3 to 56.1 mg/L generally peaking 4 or 5 days post challenge. Monocytes were elevated above the upper limit of normal on at least one time occasion post challenge in 60%, 58.3% and 75% subjects in Cohorts 1, 2 and 3 respectively. The highest values occurred in Cohort 3. Interestingly, there was a statistically significant negative correlation between lymphocyte to monocyte ratio (L:M) and clinical symptoms (P = 0.008, r = -0.725) in cohort 3. There were no other clinically significant changes in ECG results (ischaemia, pericarditis and tachycardia), respiratory function or vital signs.

### Influenza-like illness

**Figure 1:**
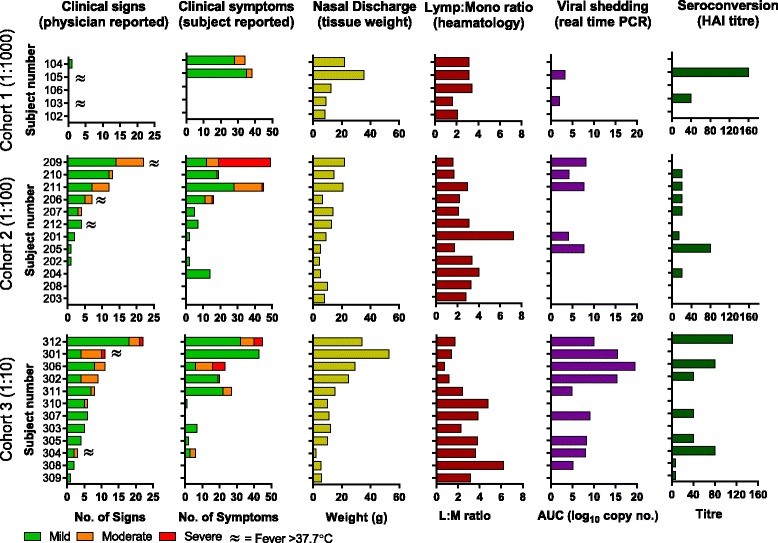
**Summary of clinical, virological and immunological responses.** Data from individual subjects is ranked per cohort according to physician-reported signs. Physician-reported signs: Data is shown as the cumulative number of clinical signs reported after influenza challenge during the quarantine period. For each subject, mild, moderate and severe clinical signs are shown as a stacked bar graph with no weighting of data applied. Fever of greater than 37.7°C is shown as a symbol next to the bar where appropriate. Subject-reported symptoms: As for clinical signs but reporting the sum of the subject-reported symptoms. Nasal discharge weight: Data is shown as the cumulative nasal discharge weight in grams. Lymp:Monocyte ratio: The lymphocyte to monocyte ratio was calculated from haematology results collected 5 days after infection. Viral shedding (rRT-PCR): The results are shown as the cumulative virus copies/mL during the quarantine period. Serology: Geometric mean HI titres to A/California/7/2009 are shown from serum samples collected at 28 days after challenge. HI tires at screening and one day prior to challenge were all negative (≤10).

There was a dose-dependent increase in subjects with at least one physician-reported sign of influenza - 20, 75 and 100% in cohorts 1 (1:1000), 2 (1:100) and 3 (1:10) respectively (Figure 1). With regard to subject-reported symptoms, there did not appear to be an overall difference between cohorts 2 and 3 in either the proportion of subjects presenting symptoms or severity of illness. Table 2 summarises the specific subject-reported symptoms and physician-reported signs. Across all cohorts, the most common symptoms were headache (44.8%), nasal stuffiness/congestion (41.4%), sore throat (37.9%) and sneezing (37.9%). The most common physician-reported signs were pharyngitis (62.1%) and nasal discharge (55.2%). There was a dose-dependent increase in pharyngitis with 20, 58.3 and 83.3% of subjects showing signs of pharyngitis in cohorts 1, 2 and 3 respectively. Most signs and symptoms indicate an upper respiratory tract infection although some symptoms and signs of a lower respiratory tract infection were recorded including new wheezes/crackles/rhonchi (6.9%) and wheezy chest (3.4%). The severity of individual signs and symptoms from the highest dose cohort (cohort 3) is shown in Table 3. All subjects in this group had signs or symptoms classified as mild, 58.3% had symptoms or signs for at least one day that were was classified as moderate and 16.7% of subjects had signs or symptoms defined as severe.

**Table 2:** **The maximum recorded symptom or sign present during the quarantine phase in cohort 3 (N = 12)**

	**Mild**		**Moderate**		**Severe**		**All**	
**Subject-reported symptoms**	**n**	**%**	**n**	**%**	**n**	**%**	**n**	**%**
Headache	4	33.3	2	16.7	1	8.3	7	58.3
Nasal stuffiness/congestion	3	25.0	2	16.7	0	0	5	41.7
Sore throat	3	25.0	2	16.7	0	0	5	41.7
Sneezing	3	25.0	3	25.0	0	0	6	50.0
Cough	2	16.7	2	16.7	1	8.3	5	41.7
Hot/feverish/chills/rigor	3	25.0	1	8.3	0	0	4	33.3
Runny nose	4	33.3	0	0	1	8.3	5	41.7
Musculoskeletal ache	1	8.3	0	0	2	16.6	3	25.0
Fatigue	2	16.7	1	8.3	0	0	3	25.0
Breathing difficulty	3	16.7	0	0	0	0	3	25.0
Diarrhoea	1	8.3	1	8.3	0	0	2	16.7
Earache	2	16.7	0	0	0	0	2	16.7
Facial or eye pain	2	16.7	0	0	1	8.3	3	25.0
Hoarseness	1	8.3	0	0	0	0	1	8.3
Nausea/vomiting	1	8.3	0	0	0	0	1	8.3
Wheezy chest	1	8.3	0	0	0	0	1	8.3
**Physician-reported signs**	**n**	**%**	**n**	**%**	**n**	**%**	**n**	**%**
Pharyngitis	10	83.3	0	0	0	0	10	83.3
Nasal discharge	3	25.0	3	25.0	2	16.6	8	66.7
Otitis	2	16.6	4	33.3	0	0	6	50.0
Sinus tenderness	2	16.6	0	0	0	0	2	16.7
New wheezes/crackles/rhonchi	1	8.3	1	8.3	0	0	2	16.7
Percussion	0	0	0	0	0	0	0	0

**Table 3:** **Summary of lab-confirmed illness**

**Inoculum dose dilution** ^**a**^	**Number of subjects**	**% rRT-PCR positive** ^**b**^	**% Sero-converted** ^**c**^	**% Clinical illness (mild)** ^**d**^	**% Clinical illness (mod-erate)** ^**e**^	**% Lab-confirmed** ***mild*** **illness** ^**f**^	**% Lab-confirmed** ***moderate*** **illness** ^**f**^
1:1000	5	20	40	40	20	20	20
1:100	12	41.6	50	75	41.6	66	41.6
1:10	12	75	50	100	58.3	75	50

^a^Neat virus was 3.5 × 10^7^ TCID_50._

^b^Assessed using a rRT-PCR assay.

^c^Seroconversion defined as a fold increase in serum HI titres of ≥4.

^d^Mild clinical illness defined as one mild physician-reported sign or subject-reported symptom.

^e^Moderate clinical illness defined as one moderate physician-reported sign or subject-reported symptom.

^f^Lab-confirmed illness defined as mild or moderate clinical illness + positive rRT-PCR result or seroconversion or both.

Seven incidences of fever defined as a temperature of >37.7°C were reported with no association to a specific dose group (Table 4). One subject in cohort 3 (subject 304) experienced a fever classified as severe and showed a maximum temperature of 39.4°C in the evening of Day 2, which was not sustained for any duration to cause clinical concern, but did peak again on Day 4 (39.4°C), before returning to normal by the morning of Day 5.

**Table 4:** **Frequencies of subject-reported symptoms, physician-reported signs and pyrexia from cohorts 1 to 3**

	**Cohort 1 (N = 5)**		**Cohort 2 (N = 12)**		**Cohort 3 (N = 12)**		**All (N = 29)**	
**Subject-reported symptoms**	**n**	**%**	**n**	**%**	**n**	**%**	**n**	**%**
Headache	2	40.0	4	33.3	7	58.3	13	44.8
Nasal stuffiness/congestion	2	40.0	5	41.7	5	41.7	12	41.4
Sore throat	1	20.0	5	41.7	5	41.7	11	37.9
Sneezing	1	20.0	4	33.3	6	50.0	11	37.9
Cough	1	20.0	4	33.3	5	41.7	10	34.5
Hot/feverish/chills/rigor	1	20.0	5	41.7	4	33.3	10	34.5
Runny nose	1	20.0	3	25.0	5	41.7	9	31.0
Musculoskeletal ache	2	40.0	3	25.0	3	25.0	8	27.6
Fatigue	2	40.0	3	25.0	3	25.0	8	27.6
Breathing difficulty	1	20.0	2	16.7	3	25.0	6	20.7
Diarrhoea	0	0.0	4	33.3	2	16.7	6	20.7
Earache	0	0.0	3	25.0	2	16.7	5	17.2
Facial or eye pain	0	0.0	2	16.7	3	25.0	5	17.2
Hoarseness	1	20.0	1	8.3	1	8.3	3	10.3
Nausea/vomiting	0	0.0	2	16.7	1	8.3	3	10.3
Wheezy chest	0	0.0	0	0.0	1	8.3	1	3.4
**Physician-reported signs**	**n**	**%**	**n**	**%**	**n**	**%**	**n**	**%**
Pharyngitis	1	20.0	7	58.3	10	83.3	18	62.1
Nasal discharge	0	0.0	8	66.7	8	66.7	16	55.2
Otitis	0	0.0	0	0.0	6	50.0	6	20.7
Sinus tenderness	0	0.0	3	25.0	2	16.7	5	17.2
New wheezes/crackles/rhonchi	0	0.0	0	0.0	2	16.7	2	6.9
Percussion	0	0.0	0	0.0	0	0.0	0	0.0
**Pyrexia**	**n**	**%**	**n**	**%**	**n**	**%**	**n**	**%**
Temperature >37.7°C	2	40.0	3	25.0	2	16.7	5	17.2

**Figure 2:**
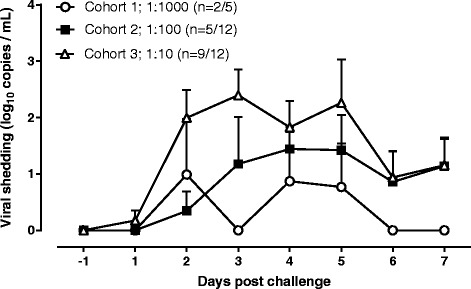
**Kinetics of Virus Shedding in infected subjects.** Viral shedding was measured in nasal wash samples collected daily during the quarantine period by means of rRT-PCR. A virtual quantification tool was applied to the rRT-PCR Ct values to generate an estimate of viral copy number [14]. The graph shows the mean copy number + SEM from subjects who became infected.

A significant correlation was measured between nasal discharge weight and subject symptom scores (r = 0.804, P = 0.003) or physician-reported sign scores (r = 0.844, P = 0.001) in the 1:10 dose group (cohort 3). The kinetics of subject symptoms scores and nasal discharge from cohort 3 showed a peak for both readouts 4 days after virus challenge (Figure 2).

### Viral shedding

Presence of virus in nasal washes, as determined by rRT-PCR, was demonstrated in 2 (40%) subjects in cohort 1 (1:1000); 5 (41.6%) subjects in cohort 2 (1:100) and 9 (75%) subjects in cohort 3 (1:10) (Figure 1). Analysis of the kinetics of viral shedding reveals that there was a dose-dependent increase in both the magnitude and duration of viral shedding (Figure 2). Of the two subjects in cohort 1 with measureable viral shedding, the median duration of shedding was 1.5 days. For cohorts 2 and 3, the median duration of viral shedding was 3 and 5.5 days respectively. The mean maximal magnitude of viral shedding for cohorts 1, 2 and 3 was 0.99, 1.44 and 2.40 log_10_ viral copies/mL of nasal wash respectively.

**Figure 3:**
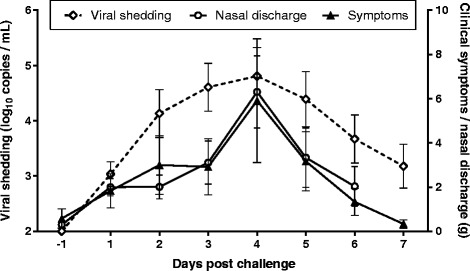
**Kinetics of clinical illness and virus infectivity.** A. The clinical symptom score, nasal discharge weight (g) and virus shedding by TCID_50_ are presented across the quarantine period for subjects in cohort 3 only (N = 12). Data is shown as mean ± SEM.

As the 1:10 was identified as the optimal dose of virus to induce both clinical illness and viral shedding, we further conducted measurements of viral infectivity using a TCID_50_ assay from this cohort only (Figure 3). Virus was detected in nasal wash samples 1 day after inoculation in 75% of subjects. Peak viral replication occurred 3 or 4 days after challenge and the geometric mean peak TCID_50_ was 10^5.16^. The median duration of viral shedding was 5 days. 25% of subjects in the 1:10 group showed evidence of residual viral shedding 7 days after challenge, the day of discharge from the quarantine unit.

### Serological responses

Volunteers were pre-screened so that all subjects had a baseline HI titre of ≤1:10 to the challenge virus. HI titres were additionally measured 28 days after challenge and a 4-fold increase in HI titre was observed in 48% of subjects with no clear association between virus dose and a positive serological response (Figure 1).

Assessing subject signs and symptoms together with laboratory assessments (rRT-PCR and/or seroconversion) we defined 20% (1:1000 group), 66% (1:100 group) and 75% (1:10 group) of subjects will mild laboratory-confirmed illness (Table 3). 20, 41.6 and 50% of subjects from cohorts 1, 2 and 3 respectively, had moderate laboratory-confirmed illness.

## Discussion

We have characterised a live, wild-type influenza A virus isolated from a human sample during the 2009 H1N1 pandemic. A low-passage GMP batch of virus was manufactured and subjected to extensive quality and adventitious agent testing, showing close identity to A/California/04/09 (H1N1) based on sequence and serological data. The virus was susceptible to neuraminidase inhibitors thereby offering a rescue therapy for future clinical use, if required. We clinically tested the infectivity of the virus in a dose-escalation study in sero-negative healthy adult volunteers. Results from the optimal cohort showed laboratory-confirmed infection in 75% of subjects defined by at least one mild or worse symptom/sign and lab-confirmed infection.

The virus induced a mild-moderate disease without such severity to be of clinical concern. At the highest dose, physician-reported signs of infection were classified as mild (100%), moderate (58%) and severe (16%). It is important to note that classifications used for mild, moderate or severe relate to influenza infection in the context of the challenge model and do not necessarily reflect medial definitions of clinical severity. It was considered that this mixed profile disease severity was suitable for use in intervention studies as this provides a window for reduction without causing overt safety concerns. Influenza challenge studies can be criticised for not mimicking the symptoms of naturally acquired influenza infection. However, whilst challenge studies are restricted to deliver an acceptable safety profile, many individuals with naturally acquired influenza infection will have a wide range of symptoms, most of which are not severe.

As of 2008, 532 of approximately 1,300 volunteers who have participated in human influenza challenge studies were administered A/H1N1 viruses [7]. Almost all of these studies enrolled healthy adult volunteers <50 years of age with low (≤1:16) pre-existing HI antibody against the challenge strain. In this study, we only observed serconversion from 48% of subjects after virus challenge. Additional testing of serum using more sensitive techniques, such as the virus microneutralisation assay, may increase the proportion of subjects with virus-specific antibody responses. However, preliminary testing using samples from cohort 3 did not show an increase in seroconversion using the microneautralisation assay (data not shown). Other studies have also reported incomplete serological responses in subjects after influenza challenge using an HAI assay [8-10]. It is possible that the kinetics of antibody production differs between subjects possibly influenced by the extent of previous exposure to influenza viruses.

The safety profile was such that physiological responses to viral infection were detected (elevated C-reactive protein and elevated monocytes, pyrexia and upper respiratory tract symptoms), but were not of such severity to cause irreversible damage or be of clinical concern. Most importantly there were no significant changes in the respiratory function of subjects and no subjects experienced any adverse cardiovascular effects. Most treatment emergent adverse events recorded (37 in total over the whole study) were predominantly considered to be related to the virus. There were no clinically-relevant changes in clinical chemistry or haematology parameters but, interestingly, there was a significant inverse correlation between lymphocyte to monocyte ratio (L:M) and clinical symptoms or scores 5 days after inoculation. These findings were aligned with a recent report suggesting that a L:M ratio of <2 correctly identified subjects infected with influenza at the time of maximal symptoms [11]. Pyrexia (>37.7°C) was measured in 7 subjects, with no association to virus dose, but was concomitant with increased C-reactive protein in 6 out of the 7 subjects.

We used two independent assessments of illness, subject-reported symptoms and physician reported signs. Physician-reported signs were considered to be a more objective assessment of illness and while the subject reported symptoms were more extensive, and typically reported for this type of clinical model, they are nevertheless subjective. There was a significant correlation between the two assessments in the highest dose cohort and therefore, combined with laboratory-tests for the presence of virus, both symptoms and signs provide useful information to define infectivity. Total nasal discharge weights closely correlated with subject symptoms and the kinetics of both measurements were well aligned. Whilst this association may not be surprising as symptoms scores include assessment of nasal discharge, the recording of nasal discharge weights provided a useful additional objective measure of clinical outcome. One limitation of this study is the lack of a control/placebo group which would allow some understanding of non-virus related symptoms or signs.

Kinetic analysis of mean symptoms in the optimal dose group showed maximal illness occurred 4 days after viral challenge, which was accompanied by peak viral shedding. This is a moderately delayed onset compared to other influenza challenge studies where peak symptom scores occur 3 days post infection and peak viral titres 2 days after infection [7] (review), [12,13]. Surprisingly, there was evidence of viral shedding on the day of discharge from the quarantine unit (7 days post challenge). For this study, these results were not available on discharge but all subjects had a negative rapid influenza test, highlighting the differences in sensitivity of the assays, and subjects were judged to be asymptomatic by the study investigator. For future studies using this challenge virus it would be prudent to include a longer quarantine period and/or include the use of antiviral agents towards the end of quarantine. Alternatively, subjects should remain in the quarantine facility until a negative PCR result is obtained.

In conclusion, the human influenza challenge model offers potential as a more controlled, rapid, and cost efficient way in which to clinically test influenza vaccines as compared to community-based studies or field trials. We have manufactured an influenza A/H1N1 virus under GMP conditions and have clinically characterised the infectivity profile of the virus as a suitable agent to use in a human influenza challenge model.

## Materials and methods

### Characterisation of A/H1N1 challenge strain

The virus was isolated from a combined nasal/throat swab collected from an otherwise healthy 3 year-old boy exhibiting typical influenza-like illness in June 2009. The boy was a prospectively enrolled participant in an Investigational Review Board-approved influenza pathogenesis study (DMID 07-0090) conducted by University of Rochester Medical Centre as part of the New York Influenza Centre of Excellence. Informed written parental consent was obtained for the use of the isolated virus. Influenza A/California/2009 (H1N1) infection was confirmed by polymerase chain reaction (PCR). Exclusion of concomitant infection with other viruses was evaluated by PCR and serology. Medical and family histories were negative at the time of the initial encounter and the boy was confirmed to have remained healthy when the family was re-contacted in 2012. An aliquot of this clinical sample was inoculated into the allantoic fluid of SPF embryonated hen eggs and expanded to GMP standards by Meridian Bioscience Inc. (OH, USA). Allantoic fluid from the 3^rd^ passage was pooled and diluted 1:5 in 37.5% sucrose phosphate glutamate as a preservative. The solution was sterile filtered and was distributed into over 2000 cryovials at 0.7 mL/vial and stored at ≤ -65°C. The virus stock had a titre of 7 × 10^7^ TCID_50_/mL. Genetic sequencing showed the virus to have >99.4% sequence identity to A/California/04/2009 HA and NA proteins (for sequence data refer to online additional material). This identity was confirmed serologically using reference sera in an HAI assay (1:1600). Extensive adventitious agents testing confirmed the absence of concomitant infectious agents. Using an in vitro NA inhibition assay, the A/H1N1 challenge strain was shown to be susceptible to oseltamivir and zanamivir with IC50 values of 0.14 and 0.24 nM respectively.

### Volunteers

The subjects were healthy adults aged 18 to 45 and serologically negative to the challenge virus (serum HAI titres ≤1:10) (Table 1). Detailed inclusion/exclusion criteria are described in clinicaltrials.gov under identifier NCT02014870 and in the online additional material. All subjects gave written informed consent to participate in the trial. The protocol and informed consent were approved by an independent ethics committee, Institutional Review Board, ZNA/OCMW, Antwerp, Belgium. The study was conducted in accordance with EU Directive 2001/20/EC and ICH GCP. The study was initiated in June 2013 and completed in October 2013.

### Clinical trial

The study was an open label, dose-ascending, non-controlled, first-time-in-human study to assess safety, tolerability and illness/infectivity profile of the influenza challenge strain. The study was conducted in purpose-built isolation unit at SGS, Antwerp, Belgium. Three cohorts were employed: the first cohort were inoculated with a 1:1000 dilution of neat virus (estimated TCID_50_ of 3.5 × 10^4^) and was increased by 10-fold for two subsequent cohorts (1:100 and 1:10 dilution of neat virus). Subjects entered the quarantine unit 24 hrs prior to virus challenge and remained for an additional 7 days.

Immediately before challenge, frozen virus was thawed rapidly in a 37°C water bath before dilution in pre-warmed (37°C) phosphate-buffered saline (PBS, Mediatech) to appropriate challenge dose. Subjects were placed in a semi-recumbent position, with the head tipped slightly back and were asked to close their palate. A total of 0.5 mL of diluted virus stock was administered intranasally to each subject (0.25 mL per nostril) using a pipette.

Symptoms were captured twice daily by self-reporting on 16 events associated with influenza illness (headache, nasal stuffiness, runny nose, sore throat, sneezing, hoarseness, earache, facial or eye pain, cough, wheezy chest, breathing difficulty, musculoskeletal ache, nausea/vomiting, feeling/hot/feverish/chills/rigor, fatigue and diarrhoea) and classified as absent, mild, moderate or severe. Classifications were defined as follows: mild: the event causes a minor discomfort, does not interfere with daily activity of the subject or does not lead to establishment of a correcting treatment; moderate: the event perturbs the usual activity of the subject and is of a sufficient severity to make the subject uncomfortable; severe: the event prevents any usual routine activity of the patient and causes severe discomfort. In addition, a more objective targeted physical exam was performed twice daily by the study physician which assessed 6 clinical parameters (nasal discharge, otitis, pharyngitis, sinus tenderness, new wheezes, crackles or rhonchi on lung auscultation and percussion) using the same classification system. Vital signs and safety laboratory tests were performed daily. Temperature was measured twice daily (oral for cohort 1 and tympanic for cohorts 2 and 3). Nasal washes were performed daily by instilling 5 mL of pre-warmed (37°C) sterile phosphate-buffered saline into each nostril and the effluent was collected, diluted 1:1 in virus transport medium (refer to WHO publication detailed below) and stored at ≤ -65°C. Daily nasal-discharge weights were determined by the collection of pre-weighed tissues in pre-weighed plastic bags assigned to each subject during each 24-hour period. On the day of discharge from the quarantine unit subjects were required to have negative result from a negative rapid influenza test (Directigen™ EZ Flu A + B, BD). Following completion of each cohort, a safety review committee reviewed symptom and viral shedding data (rRT-PCR) before recommending to proceed to the next cohort.

### Laboratory analysis

Influenza serology was performed using a hemagglutination-inhibition (HI) assay at screening (SGS Life Sciences) and at Day -1 and 29 (VisMederi srl, Siena, Italy) for all cohorts. Serum was pre-treated with receptor-destroying enzyme (RDE) II (Denka Seiken, Japan) and 2-fold serially diluted starting from a dilution of 1:10 to 1:2560 in physiological saline. Virus (A/H1N1/California/04/2009, NIBSC) was added to each well at 4 hemagglutination units (HAU)/50 μL for 1 h at room temperature, followed by 0.35% turkey red blood cells for 1 h at room temperature. Reference sheep hyperimmune antisera were provided by NIBSC.

For real-time RT-PCR (rRT-PCR), the WHO protocol “CDC protocol of real-time RT-PCR for swine influenza A(H1N1)” was followed by VisMederi srl using but only the InfA primer designed for the universal detection influenza A viruses. A specimen was considered positive for influenza A if cycle threshold (Ct) values were within Ct values of 40. A virtual quantification tool was applied to the Ct values to allow the conversion of CDC rRT-PCR Ct values to virus RNA copy number, in the absence of a standard curve run in parallel to the samples [14].

Additional analysis of nasal wash samples from cohort 3 (but not cohorts 1 or 2) was conducted using a TCID_50_ (Tissue Culture Infectious Dose 50%) assay by Viroclinics Biosciences, The Netherlands. For the TCID_50_ assay, serial ten-fold dilutions of the nasal wash samples were inoculated onto a monolayer of MDCK cells and incubated for 6 days at 37°C. The presence of virus was detected using a HI assay and the infectious virus titres are calculated using the Spearman and Kaerber method [15,16].

### Statistics and definitions

Comparisons of clinical scores with other parameters were performed by Pearson correlation. No formal sample size calculations were performed. The pre-defined definitions of attack rate were 1. Incidence of subjects experiencing at least one day with one mild subject-reported symptom or physician-reported sign and laboratory-confirmed infection or 2. Incidence of subjects experiencing at least one day with one moderate or higher subject-reported symptom or physician-reported sign and laboratory-confirmed infection. Clinical illness is defined as one mild or moderate (as appropriate) subject-reported symptom or physician-reported sign recorded from 1 to 7 days after inoculation. Daily total symptom or sign scores were calculated using the worst grade recorded on each day, for each symptom summed, using a scoring system of 0-3 relating to absent, mild, moderate and severe symptoms respectively. Total symptom or sign scores were calculated as the sum of the daily scores from post-infection to discharge.

## Additional file

Additional file 1:
**Adverse events; clinical study inclusion and exclusion criteria; sequencing data from virus HA and NA proteins.** Detailed description and table of adverse events during the quarantine period; full listing of clinical study inclusion and exclusion criteria; sequence data of challenge virus HA and NA proteins compared to A/California/4/2009 and A/California/7/2009.

## Abbreviations

TCID_50_: Tissue Culture Infectious Dose 50%
WHO: World Health Organisation
rRT-PCR: Real-time reverse transcription Polymerase Chain Reaction
L:M: Lymphocyte:Monocyte
GMP: Good Manufacturing Practice
HI: Heamagglunination inhibition
CRP: C-reactive protein
TEAE: Treatment emergent adverse event
AE: Adverse event
MDCK: Madin-Darby canine kidney
CDC: Centers for Disease and Control and Prevention
NIBSC: National Institute for Biological Standards and Control
