# Supplementary material for: Characterisation of a wild-type influenza (A/H1N1) virus strain as an experimental challenge agent in humans
Source: Virol J. 2015 Feb 3;12:13. doi: 10.1186/s12985-015-0240-5 (PMC4322439; doi:10.1186/s12985-015-0240-5)
Supplement: Additional file 1: — Adverse events; clinical study inclusion and exclusion criteria; sequencing data from virus HA and NA proteins. Detailed description and table of adverse events during the quarantine period; full listing of clinical study inclusion and exclusion criteria; sequence data of challenge virus HA and NA proteins compared to A/California/4/2009 and A/California/7/2009. [file 12985_2015_240_MOESM1_ESM.docx]

**Watson, J et al.**

**Characterisation of a wild-type influenza (A/H1N1) virus strain as an experimental challenge agent in humans.**

**Additional Material.**

Adverse events

No subjects withdrew from the study and no serious adverse events were recorded. Viral challenge was expected to induce symptoms/signs typical of influenza infection and were captured as infectivity endpoints in the study and not as adverse events, unless they were graded as severe by either the subject or investigator. Supplementary Table 1 summarises all the treatment-emergent adverse events (TEAE) that were reported during the quarantine phase. During the challenge period, 3 (60.0%) subjects of Cohort 1, 6 (50.0%) subjects of Cohort 2, and 5 (41.7%) subjects of Cohort 3 had reported at least one TEAE. In total 14 subjects (48.3%) reported 34 events. Most TEAEs were mild or moderate in severity. No severe TEAEs were reported in Cohort 1, one severe TEAE of cough was reported for 1 subject (8.3%) in Cohort 2 and four severe AEs (back pain, pyrexia, headache and cough) were reported for 3 subjects (25.0) in Cohort 3. No treatment-related TEAEs were reported in Cohort 1. Treatment-related TEAEs were reported in 4 subjects (33.3%) each in Cohorts 2 and 3. No TEAEs were reported during the safety follow up period (28 days post challenge).

| **System Organ Class**  Adverse event, n (%) | **Cohort 1**  **(1:1000)**  N = 5 | **Cohort 2**  **(1:100)**  N = 12 | **Cohort 3**  **(1:10)**  N = 12 |
| --- | --- | --- | --- |
| **Any TEAE** | **3 (60.0)** | **6 (50.0)** | **5 (41.7)** |
| **Musculoskeletal and Connective Tissue Disorders** | **3 (60.0)** | **2(16.7)** | **4 (33.3)** |
| Back pain | 3 (60.0) | 0 | 3 (25.0) |
| Myalgia | 0 | 2 (16.7) | 2 (16.7) |
| **Nervous System Disorders** | **0** | **2 (16.7)** | **3 (25.0)** |
| Dizziness postural | 0 | 0 | 1 (8.3) |
| Headache | 0 | 2(16.7) | 2 (16.7) |
| **General Disorders and Administration Site Conditions** | **0** | **2 (16.7)** | **2 (16.7)** |
| Facial pain | 0 | 1 (8.3) | 0 |
| Fatigue | 0 | 1 (8.3) | 0 |
| Pyrexia | 0 | 2 (16.7) | 2 (16.7) |
| **Injury, Poisoning And Procedural Complications** | 0 | **2 (16.7)** | 0 |
| Catheter site pain | 0 | 1 (8.3) | 0 |
| Joint injury | 0 | 1 (8.3) | 0 |
| Wound | 0 | 1 (8.3) | 0 |
| **Infections And Infestations** | **0** | **0** | **2 (16.7)** |
| Rhinitis | 0 | 0 | 2 (16.7) |
| **Respiratory, Thoracic And Mediastinal Disorders** | **0** | **1 (8.3)** | **2 (16.7)** |
| Cough | 0 | 1 (8.3) | 1 (8.3) |
| Dyspnoea | 0 | 1 (8.3) | 0 |
| Nasal congestion | 0 | 1 (8.3) | 0 |
| Rhinorrhoea | 0 | 1 (8.3) | 1 (8.3) |
| Sneezing | 0 | 1 (8.3) | 0 |
| **Gastrointestinal Disorders** | **0** | **1 (8.3)** | **0** |
| Diarrhoea | 0 | 1(8.3) | 0 |
| **Supplementary Table 1. Adverse events.** N = number of subjects per cohort; n = number of subjects with this observation at least once across the study period | | | |

Clinical study inclusion and exclusion criteria

Inclusion Criteria

Subjects meeting all of the following criteria were eligible to participate in the study:

1. Male or female aged 18-45 years inclusive.
2. Able to give written informed consent to participate.
3. Healthy, as determined by medical history, physical examination, vital signs, 12-lead ECG, and clinical safety laboratory examinations at baseline, asdetermined by the Investigator.
4. Absent or low levels of detectable pre-existing antibodies to influenza virussubtypes, including as a minimum the challenge strain, as determined by an HAI titre of ≤ 10 prior to challenge.
5. Non-habitual smoker (habitual smokers are persons who smoke more thancigarettes or other tobacco products on a weekly basis) and agree to not use tobacco products during participation in the study.
6. Females should fulfil one of the following criteria:

*i.* At least one year post-menopausal;

*ii.* Surgically sterile;

*iii.* Will use oral, implantable, transdermal or injectable contraceptives for 30 days prior to administration of the A/California/H1N1 2009 virus until the follow-up visit is performed.

*iv.* Use another reliable form of contraception approved by the Investigator (e.g., intrauterine device, female condom, diaphragm with spermicide, cervical cap, use of condom by the sexual partner or a sterile sexual partner) from the time of screening until the follow-up visit is performed.

*v.* Women of childbearing potential must have a negative serum pregnancy test at screening and a negative urine pregnancy test on Day -1.

1. Comprehension of the study requirements, expressed availability for the required study period, and ability to be quarantined for up to 10 days and to attend the scheduled follow-up visit.
2. Negative alcohol and urine drug screening tests prior to entering quarantine.
3. Being willing to adhere to the prohibitions and restrictions specified in the clinical study protocol

Exclusion Criteria

Subjects meeting one or more of the following criteria were excluded from participation in the study:

1. Prior receipt of any influenza vaccine for the previous 2 years.
2. Significant adulthood history of seasonal hay fever or a seasonal allergic rhinitis or perennial allergic rhinitis or chronic or nasal or sinus condition such as chronic sinusitis.
3. Abnormal nasal structure including septal deviation and nasal polyps.
4. Suffering from asthma, bronchiectasis, emphysema, chronic obstructive pulmonary disease or any other chronic lung disease.
5. Pregnant or who is breast feeding.
6. Diastolic BP < 50 or > 90 mmHg, a systolic BP < 100 or > 150 mmHg, a pulse < 50 or > 100 bpm after resting for 5 min.
7. Current use or use within the last 7 days of intranasal corticosteroids.
8. Presence of significant uncontrolled medical, neurological or psychiatric illness (acute or chronic) as assessed by the Investigator. This includes, but is not limited to, institution of new surgical or medical treatment (for a chronic condition), or a significant dose alteration for uncontrolled symptoms or drug toxicity within 3 months of screening and reconfirmed on Day -1 prior to challenge.
9. Positive serology for HIV-1 or HIV-2, or HBsAg or HCV antibodies.
10. Cancer or treatment for cancer, within 3 years, excluding basal cell carcinoma of the skin, which is allowed.
11. Presence of immunosuppression or any medical condition that may be associated with impaired immune responsiveness, including, but not limited to, diabetes mellitus inflammatory bowel disease.
12. Presently receiving (or history of receiving) or during the preceding 3-month period prior to screening, any medications or other treatments that may adversely affect the immune system such as allergy injections, immune globulin, interferon, immunomodulators, cytotoxic drugs or other drugs known to be frequently associated with significant major organ toxicity, or systemic corticosteroids (oral or injectable) azathioprine or mercaptopurine. Topical corticosteroids except intranasal will be allowed.
13. Anticipated presence of a household contact with documented severe immunosuppression (as defined by CD4 < 200/mm³ or an absolute neutrophil count < 1500/mm³), either as a result of disease and/or therapy.
14. Anticipated presence of a household contact age 5 years or younger, within 2 weeks following challenge.
15. Anticipated presence of a household contact age 65 years or older, within 2 weeks following challenge.
16. Current professional activity as a carer or healthcare workers who will return to work within 2 weeks following challenge.
17. Anticipated presence of a pregnant household contact, within 2 weeks following challenge.
18. History of anaphylactic type reaction to egg or egg protein.
19. History of Guillain-Barré syndrome.
20. History of drug or chemical abuse in the year before the study.
21. Receipt of any investigational virus product or nonregistered drug within the 30 days prior to challenge or currently enrolled in any investigational drug study or intends to enrol in such a study within the ensuing study period.
22. Receipt of blood or blood products 6 months prior to challenge or planned administration during the study period.
23. Blood donation in the last 12 weeks.
24. Acute disease within 72 h prior to challenge, defined as the presence of a moderate or severe illness with or without fever (as determined by the Investigator through medical history and physical examination), or presence of a fever ≥ 38ºC oral.
25. Elevated white cell count above 10.5 x 10^9^/L or an absolute neutrophil count above 7.5 x 10^9^/L.
26. Any condition that, in the opinion of the Investigator, might interfere with the primary study objective.
27. Habitual smoker (habitual smokers are persons who smoke more than 4 cigarettes or other tobacco products on a weekly basis).

Sequence alignment

Viral RNA was extracted using a Qiagen QIamp Viral RNA mini kit and subsequently submitted to DNAse treatment. Following cycling probe real-time PCR and sequencing of the hemagglutinin (HA) and neuraminidase (NA) region, sequence analysis were carried out to classify and confirm the identity of the investigational challenge virus strain.

Primers used for the PCR amplification of the cDNA of HA were selected against a Genbank reference for which the entire coding sequence of the gene is available (accession JF915184.1; Influenza A virus (A/California/04/2009 [H1N1]) segment 4 HA gene, complete coding region). Figure 1 depicts the cDNA sequence corresponding to the HA gene segment (1623 nucleotides) of the challenge virus

Primers used for the PCR amplification of the cDNA of NA were selected against a Genbank reference for which the entire coding sequence of the gene is available (accession JF915186.1; Influenza A virus (A/California/04/2009(H1N1)) segment 6 neuraminidase (NA) gene, complete coding region) Figure 2 depicts the cDNA sequence corresponding to the NA gene segment (1172 nucleotides) of the challenge virus.

The combined sequence of two cDNA fragments (HA1 and HA2) corresponded to a total of 1623 nucleotides and representing 95.6% of the coding regions of HA gene (Figure 1) was used to determine the strain of the challenge agent. The sequence of the cDNA fragment is 99.7% identical to a A/California/2009 consensus sequence corresponding to isolates that have circulated in humans from March 2009 to June 2009 and is 99.4% and 99.6% identical to A/California/04/2009 [H1N1] and A/California/07/2009 [H1N1] reference strains respectively (see Figure 1).

The sequence of a cDNA fragment obtained by PCR corresponding to 1172 nucleotides (Figure 2) and representing 83% of the coding regions of NA gene was used to also determine the strain of the challenge agent. The sequence of the cDNA fragment is 99.8% identical to a A/California/2009 consensus sequence corresponding to isolates that have circulated in humans from March 2009 to June 2009 and is 99.6% and 99.5% identical to the A/California/04/2009 [H1N1] and A/California/07/2009 [H1N1] reference strains respectively (Figure 2).

Figure 1. Alignment of the sequenced HA gene segment with A/California/07/2009 (H1N1) and A/California/04/2009 (H1N1).

1 100

HA AA CAATTCAACA GACACTGTAG

CAL04 ATGAAGGCAA TACTAGTAGT TCTGCTATAT ACATTTGCAA CCGCAAATGC AGACACATTA TGTATAGGTT ATCATGCG.. .......... ..........

CAL07 ATGAAGGCAA TACTAGTAGT TCTGCTATAT ACATTTGCAA CCGCAAATGC AGACACATTA TGTATAGGTT ATCATGCG.. .......... ..........

101 200

HA ACACAGTACT AGAAAAGAAT GTAACAGTAA CACACTCTGT TAACCTTCTA GAAGACAAGC ATAACGGGAA ACTATGCAAA CTAAGAGGGG TAGCCCCATT

CAL04 .......... .......... .......... .......... .......... .......... .......... .......... .......... ..........

CAL07 .......... .......... .......... .......... .......... .......... .......... .......... .......... ..........

201 300

HA GCATTTGGGT AAATGTAACA TTGCTGGCTG GATCCTGGGA AATCCAGAGT GTGAATCACT CTCCACAGCA AGCTCATGGT CCTACATTGT GGAAACATCT

CAL04 .......... .......... .......... .......... .......... .......... .......... .......... .......... .......C..

CAL07 .......... .......... .......... .......... .......... .......... .......... .......... .......... .......C..

301 400

HA AGTTCAGACA ATGGAACGTG TTACCCAGGA GATTTCATCG ATTATGAGGA GCTAAGAGAG CAATTGAGCT CAGTGTCATC ATTTGAAAGG TTTGAGATAT

CAL04 .......... .......... .......... .......... .......... .......... .......... .......... .......... ..........

CAL07 .......... .......... .......... .......... .......... .......... .......... .......... .......... ..........

401 500

HA TCCCCAAGAC AAGTTCATGG CCCAATCATG ASTCGAACAA AGGTGTAACG GCAGCATGTC CTCATGCTGG AGCAAAAAGC TTCTACAAAA ATTTAATATG

CAL04 .......... .......... .......... .C........ .......... .......... .......... .......... .......... ..........

CAL07 .......... .......... .......... .C........ .......... .......... .......... .......... .......... ..........

501 600

HA GCTAGTTAAA AAAGGGAATT CATACCCAAA GCTCAGCAAA TCCTACATTA ATGATAAAGG GAAAGAAGTC CTCGTGCTAT GGGGCATTCA CCATCCATCT

CAL04 .......... .....A.... .......... .......... .......... .......... .......... .......... .......... ..........

CAL07 .......... .....A.... .......... .......... .......... .......... .......... .......... .......... ..........

601 700

HA ACTAGTGCTG ACCAACAAAG TCTCTATCAG AATGCAGATG CATATGTTTT TGTGGGGACA TCAAGATACA GCAAGAAGTT CAAGCCGGAA ATAGCAATAA

CAL04 .......... .......... .......... .........A .......... .......T.. .......... .......... .......... ..........

CAL07 .......... .......... .......... .......... .......... .......T.. .......... .......... .......... ..........

701 800

HA GACCCAAAGT GAGGGATCAA GAAGGGAGAA TGAACTATTA CTGGACACTA GTAGAGCCGG GAGACAAAAT AACATTCGAA GCAACTGGAA ATCTAGTGGT

CAL04 .......... .......... .......... .......... .......... .......... .......... .......... .......... ..........

CAL07 .......... .......... .......... .......... .......... .......... .......... .......... .......... ..........

801 900

HA ACCGAGATAT GCATTCGCAA TGGAAAGAAA TGCTGGATCT GGTATTATCA TTTCAGATAC ACCAGTCCAC GATTGCAATA CAACTTGTCA GACACCCAAG

CAL04 .......... .......... .......... .......... .......... .......... .......... .......... .......... A.........

CAL07 .......... .......... .......... .......... .......... .......... .......... .......... .......... A.........

901 1000

HA GGTGCTATAA ACACCAGCCT CCCATTTCAG AATATACATC CGATCACAAT TGGAAAATGT CCAAAATATG TAAAAAGCAC AAAATTGAGA CTGGCCACAG

CAL04 .......... .......... .......... .......... .......... .......... .......... .......... .......... ..........

CAL07 .......... .......... .......... .......... .......... .......... .......... .......... .......... ..........

1001 1100

HA GATTGAGGAA TGTCCCGTCT ATTCAATCTA GAGGCCTATT TGGGGCCATT GCCGGTTTCA TTGAAGGGGG GTGGACAGGG ATGGTAGATG GATGGTACGG

CAL04 .......... .A........ .......... .......... .......... .......... .......... .......... .......... ..........

CAL07 .......... .A........ .......... .......... .......... .......... .......... .......... .......... ..........

1101 1200

HA TTATCACCAT CAAAATGAGC AGGGGTCAGG ATATGCAGCC GACCTGAAGA GCACACAGAA TGCCATTGAC GAGATTACTA ACAAAGTAAA TTCTGTTATT

CAL04 .......... .......... .......... .......... .......... .......... .......... .......... .......... ..........

CAL07 .......... .......... .......... .......... .......... .......... .......... .......... .......... ..........

1201 1300

HA GAAAAGATGA ATACACAGTT CACAGCAGTA GGTAAAGAGT TCAACCACCT GGAAAAAAGA ATAGAGAATT TAAATAAAAA AGTTGATGAT GGTTTCCTGG

CAL04 .......... .......... .......... .......... .......... .......... .......... .......... .......... ..........

CAL07 .......... .......... .......... .......... .......... .......... .......... .......... .......... ..........

1301 1400

HA ACATTTGGAC TTACAATGCC GAACTGTTGG TTCTATTGGA AAATGAAAGA ACTTTGGACT ACCACGATTC AAATGTGAAG AACTTATATG AAAAGGTAAG

CAL04 .......... .......... .......... .......... .......... .......... .......... .......... .......... ..........

CAL07 .......... .......... .......... .......... .......... .......... .......... .......... .......... ..........

1401 1500

HA AAGCCAGTTA AAAAACAATG CCAAGGAAAT TGGAAACGGC TGCTTTGAAT TTTACCACAA ATGCGATAAC ACGTGCATGG AAAGTGTCAA AAATGGGACT

CAL04 .......C.. .......... .......... .......... .......... .......... .......... .......... .......... ..........

CAL07 .......C.. .......... .......... .......... .......... .......... .......... .......... .......... ..........

1501 1600

HA TATGACTACC CAAAATACTC AGAGGAAGCA AAATTAAACA GAGAAGAAAT AGATGGGGTA AAGCTGGAAT CAACAAGGAT TTACCAGATT TTGGCGATCT

CAL04 .......... .......... .......... .......... .......... .......... .......... .......... .......... ..........

CAL07 .......... .......... .......... .......... .......... .......... .......... .......... .......... ..........

1601 1700

HA ATTCAACTGT CGCCAGTTCA TTGGTACTGG TAGTCTCCCT GGGGGCAATC AGTTTCTGGA TGTGCTCTAA TGGGTCTCTA CAGTGTAGAA TATGTATTTA

CAL04 .......... .......... .......... .......... .......... .......... .......... .......... .......... ..........

CAL07 .......... .......... .......... .......... .......... .......... .......... .......... .......... ..........

1701

HA A

CAL04 .

CAL07 .

|  | Position 100 | Position 214 | Position 220 | Position 338 |
| --- | --- | --- | --- | --- |
| A/California/07/2009(H1N1) | S→P | - | T→S | V→I |
| A/California/04/2009(H1N1) | S→P | A→T | T→S | V→I |

**Supplementary Table 2.** Possible amino acid changes in HA.

Figure 2. Alignment of the sequenced NA segment with A/California/07/2009 (H1N1) and A/California/04/2009 (H1N1).

1 100

NA CCATT GGTTCGGTCT GTATGACAAT TGGAATGGCT AACTTAATAT TACAAATTGG AAACATAATC TCAATATGGA

CAL04 ATGAATCCAA ACCAAAAGAT AATAA..... .......... .......... .......... .......... .......... .......... ..........

CAL07 ATGAATCCAA ACCAAAAGAT AATAA..... .......... .......... .......... .......... .......... .......... ..........

101 200

NA TTAGCCACTC AATTCAACTT GGGAATCAAA ATCAGATTGA AACATGCAAT CAAAGCGTCA TTACTTATGA AAACAACACT TGGGTAAATC AGACATATGT

CAL04 .......... .......... .......... .......... .......... .......... .......... .......... .......... ..........

CAL07 .......... .......... .......... .......... .......... .......... .......... .......... .......... ..........

201 300

NA TAACATCAGC AACACCAACT TTGCTGCTGG ACAGTCAGTG GTTTCCGTGA AATTAGCGGG CAATTCCTCT CTCTGCCCTG TTAATGGATG GGCTATATAC

CAL04 .......... .......... .......... .......... .......... .......... .......... .......... ...G...... ..........

CAL07 .......... .......... .......... .......... .......... .......... .......... .......... ...G...... ..........

301 400

NA AGTAAAGACA ACAGTATAAG AATCGGTTCC AAGGGGGATG TGTTTGTCAT AAGGGAACCA TTCATATCAT GCTCCCCCTT GGAATGCAGA ACCTTCTTCT

CAL04 .......... .....G.... .......... .......... .......... .......... .......... .......... .......... ..........

CAL07 .......... .....G.... .......... .......... .......... .......... .......... .......... .......... ..........

401 500

NA TGACTCAAGG GGCCTTGCTA AACGACAAAC ATTCCAATGG AACCATTAAA GACAGGAGCC CATATCGAAC CCTAATGAGC TGTCCTATTG GTGAAGTTCC

CAL04 .......... .......... ..T....... .......... .......... .......... .......... .......... .......... ..........

CAL07 .......... .......... ..T....... .......... .......... .......... .......... .......... .......... ..........

501 600

NA CTCTCCATAC AACTCAAGAT TTGAGTCAGT CGCTTGGTCA GCAAGTGCTT GTCATGATGG CATCAATTGG CTAACAATTG GAATTTCTGG CCCAGACAAT

CAL04 .......... .......... .......... .......... .......... .......... .......... .......... .......... ..........

CAL07 .......... .......... .......... .......... .......... .......... .......... .......... .......... ..........

601 700

NA GGGGCAGTGG CTGTGTTAAA GTACAACGGC ATAATAACAG ACACTATCAA GAGTTGGAGA AACAATATAT TGAGAACACA AGAGTCTGAA TGTGCATGTG

CAL04 .......... .......... .......... .......... .......... .......... .......... .......... .......... ..........

CAL07 .......... .......... .......... .......... .......... .......... .......... .......... .......... ..........

701 800

NA TAAATGGTTC TTGCTTTACT GTAATGACCG ATGGACCAAG TGATGGACAG GCCTCATACA AGATCTTCAG AATAGAAAAG GGAAAGATAG TCAAATCAGT

CAL04 .......... .......... .......... .......... .A........ .......... .......... .......... .......... ..........

CAL07 .......... .......... .......... .......... .A........ .......... .......... .......... .......... ..........

801 900

NA CGAAATGAAT GCCCCTAATT ATCACTATGA GGAATGCTCC TGTTATCCTG ATTCTAGTGA AATCACATGT GTGTGCAGGG ATAACTGGCA TGGCTCGAAT

CAL04 .......... .......... .......... .......... .......... .......... .......... .......... .......... ..........

CAL07 .......... .......... .......... .......... .......... .......... .......... .......... .......... ..........

901 1000

NA CGACCGTGGG TGTCTTTCAA CCAGAATCTG GAATATCAGA TAGGATACAT ATGCAGTGGG ATTTTCGGAG ACAATCCACG CCCTAATGAT AAGACAGGCA

CAL04 .......... .......... .......... .......... .......... .......... .......... .......... .......... ..........

CAL07 .......... .......... .......... .......... .......... .......... .......... .......... .......... ..........

1001 1100

NA GTTGTGGTCC AGTATCGTCT AATGGAGCAA ATGGAGTAAA AGGATTTTCA TTCAAATACG GCAATGGTGT TTGGATAGGG AGAACTAAAA GCATTAGTTC

CAL04 .......... .......... .......... .......... ...G...... .......... .......... .......... .......... ..........

CAL07 .......... .......... .......... .......... ...G...... .......... .......... .......... .......... ..........

1101 1200

NA AAGAAACGGT TTTGAGATGA TTTGGGATCC GAACGGATGG ACTGGGACAG ACAATAACTT CTCAATAAAG CAAGATATCG TAGGAATAAA TGAGTGG

CAL04 .......... .......... .......... .......... .......... .......... .......... .......... .......... .......TCA

CAL07 .......... .......... .......... .......... .......... .......... .......... .......... .......... .......TCA

1201 1300

NA

CAL04 GGATATAGCG GGAGTTTTGT TCAGCATCCA GAACTAACAG GGCTGGATTG TATAAGACCT TGCTTCTGGG TTGAACTAAT CAGAGGGCGA CCCAAAGAGA

CAL07 GGATATAGCG GGAGTTTTGT TCAGCATCCA GAACTAACAG GGCTGGATTG TATAAGACCT TGCTTCTGGG TTGAACTAAT CAGAGGGCGA CCCAAAGAGA

1301 1400

NA

CAL04 ACACAATCTG GACTAGCGGG AGCAGCATAT CCTTTTGTGG TGTAAACAGT GACACTGTGG GTTGGTCTTG GCCAGACGGT GCTGAGTTGC CATTTACCAT

CAL07 ACACAATCTG GACTAGCGGG AGCAGCATAT CCTTTTGTGG TGTAAACAGT GACACTGTGG GTTGGTCTTG GCCAGACGGT GCTGAGTTGC CATTTACCAT

1401 1410

NA

CAL04 TGACAAGTAA

CAL07 TGACAAGTAA

|  | Position 95 | Position 106 | Position 248 |
| --- | --- | --- | --- |
| A/California/07/2009(H1N1) | N→S | I→V | D→N |
| A/California/04/2009(H1N1) | N→S | I→V | D→N |

**Supplementary Table 3.** Possible amino acid changes in NA.
